# Soybean Oil-Based Biopolymers Induced by Nonthermal Plasma to Enhance the Dyeing of Para-Aramids with a Cationic Dye

**DOI:** 10.3390/polym14030628

**Published:** 2022-02-06

**Authors:** Caleb Metzcar, Xiaofei Philip Ye, Toni Wang, Christopher J. Doona

**Affiliations:** 1 Department of Food Science, The University of Tennessee, Knoxville, TN 37996, USA; cmetzcar@vols.utk.edu (C.M.); twang46@utk.edu (T.W.); 2 Department of Biosystems Engineering and Soil Science, The University of Tennessee, Knoxville, TN 37996, USA; 3 U.S. Army Combat Capabilities Development Command—Soldier Center, Natick, MA 01760, USA; doonac@mit.edu; 4 Massachusetts Institute of Technology—Institute for Soldier Nanotechnologies, 77 Massachusetts Ave NE47-4F, Cambridge, MA 02139, USA

**Keywords:** para-aramid, nonthermal plasma, ambient air, soybean oil, acrylic acid, acrylated epoxidized soybean oil, cationic dye

## Abstract

To overcome the recalcitrance of para-aramid textiles against dyeing, this study demonstrated that increasing the functionalities of soybean oil applied to the surface of para-aramids followed by a nonthermal plasma (NTP) treatment improved the dyeing color strength compared with the use of soybean oil alone, and that dyeing occurred through covalent bonding. Particularly, compared with the pretreatment using soybean oil that obtained the highest color strength of 3.89 (as K/S value determined from spectral analysis of the sample reflectance in the visible range), the present pretreatments with either acrylated epoxidized soybean oil (AESO) or a mixture of acrylic acid and soybean oil (AA/Soy) achieved K/S values higher than nine (>9.00). The NTP treatment, after the AESO or AA/Soy pretreatment, was essential in inducing the formation of a polymerized network on the surface of para-aramids that bonded the dye molecules and generating covalent bonds that anchored the polymerized network to the para-aramids, which is difficult to achieve given the high crystallinity and chemical inertness of para-aramids. As an important economic consideration, the sequential experimentation method demonstrated that a simple mixture of AA/Soy could replace the expensive AESO reagent and render a comparable performance in dyeing para-aramids. Among the auxiliary additives tested with the AESO and AA/Soy pretreatments followed by NPT treatment in this study, Polysorbate 80 as a surfactant negatively affected the dyeing, benzyl alcohol as a swelling agent had minimal effect, and NaCl as an electrolyte showed a positive effect. The dyeing method developed in this study did not compromise the strength of para-aramids.

## 1. Introduction

The invention of aramids with commercial names such as Kevlar^®^ (para-aramid) and Nomex^®^ (meta-aramid) brought about a synthetic fiber with high strength-to-weight ratio, low elongation to break, superior heat and flame resistance, high cut resistance, and excellent ballistic properties [1]. Since the first commercial use of Kevlar^®^ in the early 1970s as a replacement for steel in racing tires, aramid materials have been used in an increasing number of diverse applications. Examples include textiles for protective clothing (e.g., flame-resistant apparel) and body armor (e.g., bullet-proof vests, helmets, and puncture-resistant correctional wear), sportswear, and reinforced composites (e.g., brake pads, gaskets, hot-air filters, industrial belts and ropes, and strength member in fiber optics) [2]. It is highly desirable to incorporate aramids into additional applications for protective clothing, but these efforts are complicated by the difficulty associated with durably dyeing or printing aramids.

Between the two major types of aramids, meta-aramids and para-aramids, the meta-aramid fibers consist of poly(m-phenylene isophthalamide) that binds via meta-linked aromatic rings to result in a semi-crystalline fiber with the molecular chain oriented along the fiber axis, while the building-blocks of para-aramids are poly(p-phenylene terephthalamide) with stiff para-linked aromatic rings and densely arranged hydrogen bond donors and acceptors throughout their backbones [3]. This inherent molecular rigidity of para-aramids, combined with strong intermolecular hydrogen bonding interactions, enables the molecules to achieve excellent alignment with their neighbors, resulting in a highly anisotropic unit cell consisting of covalent bonds, hydrogen bonds, and van der Waals interactions along each fundamental axis, forming a highly crystalline structure [4]. The high degree of crystallinity and chemical inertness makes it difficult to dye para-aramids with conventional dyeing methods, because para-aramids cannot entrap or bind dye molecules.

There are a number of methods reported for dyeing meta-aramids, but few methods reported for dyeing para-aramids (especially continuous filament para-aramids) without using harsh chemicals that can damage the dyed materials and sacrifice mechanical strength [5,6,7]. Typically, aramid filaments/yarns are dyed to a single color using solution dyeing methods, a process in which acid-tolerant colorant is added to the polymer dope at the time of aramid filament production, and the dyed filaments are subsequently woven into usable fabrics with only a limited number of color choices available by these methods [8]. While it is highly desirable to incorporate aramids into new applications for protective clothing or outerwear, for example, the difficulty in durably dyeing or printing aramids is a barrier to using aramids in these applications. New methods of dyeing para-aramids that avoid the use of harsh or environmentally unsafe chemicals would be advantageous for these applications.

Our previous studies [9,10] demonstrated that pretreatment with soybean oil followed by nonthermal plasma (NTP) treatment enabled dyeing para-aramids to significantly high color strength without other chemical additives; and this new method is compatible with both a disperse dye and a basic dye, showing the potential of this method replacing the environmentally unfriendly chemicals in current dyeing practices with renewable, environmentally friendly materials to improve the dyeing of para-aramid fabrics. The proposed mechanism for improved dyeing is that the soybean oil diffuses onto woven fabric and adsorbs onto the surfaces of yarns and fibers, and the subsequent NTP treatment induces the formation of a polymerized network in situ, enabling dyeing to a higher color strength. The color strength in term of K/S value, determined from spectral analysis of the sample reflectance in the visible range, increased to 3.89 from ~1 of the untreated samples. Along this line of sustainable dyeing method, in this study, we aim at further improving the dyeing color strength and colorfastness by deriving more functionalities of soybean oil on the surface of para-aramids.

Acrylated epoxidized soybean oil (AESO) synthesized from soybean oil has already occupied a significant share of market as a “green” alternative to petroleum-based epoxy resins, plasticizers, and pre-polymers [11]. It contains three highly reactive functionalities of double (C=C) bonds, –OH groups, and epoxy rings. The C=C bond in AESO is capable of self-polymerizing and copolymerizing with other components via a free radical initiation (including UV and NTP, which generate both UV and free radical species), forming a network with ample functionalities to bind with dyes [12]. However, the current method of soybean oil epoxidation followed by acrylate addition for AESO production is a tedious process, rendering a high price for AESO. We hypothesized that pretreating para-aramids with a simple mixture of soybean oil and acrylic acid followed by NTP treatment would induce a similar polymerized network in situ to enhance the dyeing. Importantly, acrylic acid can also be produced from renewable glycerol, which is a co-product of massive biodiesel production, mainly from soybean oil in the U.S. Acrylic acid can be produced via the intermediate of glycerol dehydration to acrolein, and this strategy has received much attention because it appears to be one of the most promising ways to valorize glycerol [13,14,15].

Furthermore, because the dyeing industry often uses auxiliary chemical additives of surfactants, electrolytes, and swelling agents to improve dyeing performance [16,17,18], the effects of Polysorbate 80 as a surfactant, sodium chloride (NaCl) as an electrolyte, and benzyl alcohol as a swelling agent, were also examined in our new method.

## 2. Materials and Methods

The following section describes all materials and methods used in this study. Overall, a series of experiments, designed as sequential experimentation, directed this study and demonstrated the potential for this new method to improve the dyeing of para-aramids. The experimental design and analysis were conducted using Design-Expert software (version 6, Stat-Ease, Inc., Minneapolis, MN, USA). Because many experimental factors were involved, our design of experiments emphasized the sequential use of two-level factorial designs to identify critical factors for improving dyeing, and the sequential assembly of second-order designs to elucidate the nature of the response surface in the improved formulations and process conditions.

### 2.1. Materials

The para-aramid fabrics used in this study are made of tightly woven 300 Denier continuous filament and were provided by the U.S. Army Development Command—Soldier Center (Natick, MA, USA). This highly crystalline material has been proven undyeable by conventional methods using either a cationic dye or a disperse dye, even with nonthermal plasma surface treatment, but were dyed using soybean oil/NTP pretreatments [9,10]. Blue cationic dye (Victoria Blue R, CAS Number 2185-86-6), Disperse Red 1 acrylate dye (CAS Number 13695-46-0), AESO (CAS Number 91722-14-4), acrylic acid (CAS Number 79-10-7), and ethyl acetate (CAS Number 141-78-6) were purchased from Sigma-Aldrich (St. Louis, MO, USA). Polysorbate 80 (a nonionic surfactant with the common name TWEEN 80 and the IUPAC name polyoxyethylene (20) sorbitan monooleate) and benzyl alcohol (a common swelling agent) were purchased from Chem Center @ Amazon.com. Commercial food-grade refined soybean oil (typically consisting of about 23% monounsaturated fat, 58% polyunsaturated fat, and 15% saturated fat) and laundry detergent (ECOS^®^ plus stain-fighting enzymes) were purchased from a local supermarket. All materials in this study were used as-is and without further purification.

### 2.2. Pretreatment and NTP Treatment

Each para-aramid fabric sample was cut into approximately one square inch swatch. Depending on the experimental design, the pretreatment was conducted by submerging the samples in a solution of AESO or a mixture of acrylic acid and soybean oil (denoted as AA/Soy hereafter) in ethyl acetate in a sealed beaker for a designated time. Then, the samples were taken out, placed on a paper towel, and pressed with a roller two times to remove excessive pretreatment liquid, followed by NTP treatment if required by the experimental design.

**Figure 1:**
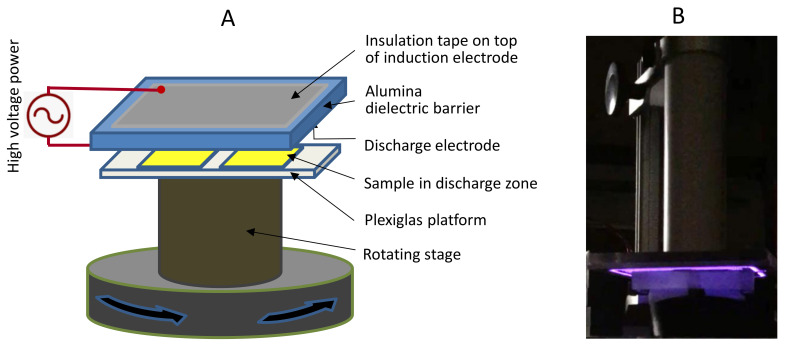
(**A**) Schematic of NTP treatment and (**B**) Photo image showing glowing surface discharge on top of samples in dark background.

The NTP treatment of the fabric samples was carried out using an in-house made surface dielectric barrier discharge (SDBD) apparatus consisting of two electrodes separated by a 108 × 95 mm alumina dielectric plate with a thickness of 1 mm (Figure 1). The alumina plate has an induction electrode made of a rectangular copper tape embedded in insulation tape on its top and a discharge electrode made of 17 interconnected tungsten strips on its lower surface. Teflon-coated aramids have been made wettable after a 30 s exposure to this SDBD. Compared with volume dielectric barrier discharge (VDBD), SDBD generates a higher density of micro-discharges that are limited to the surface of the sample, and thereby avoids pin-holing that VDBD caused in para-aramids by the hot electron bombardment, weakening the fibers. The feedgas for the SDBD is ambient air, and the power for the SDBD was a 9.2 kV sinusoidal high voltage source tuned to a resonance frequency of 23.2 kHz.

Para-aramid samples were placed on top of a Plexiglas platform mounted on a rotating stage, and the SDBD plate was lowered via an adjustable stand to 1 mm above and parallel to the sample to ensure uniform treatment of the sample. Surface emissions, reactive oxygen and nitrogen species (RONS), and other radicals generated in the plasma interacted with the para-aramid samples. At completion of the NTP treatment of a specified time, the power to the SBSD was turned off and the sample was removed immediately thereafter.

### 2.3. Dyeing Experimental Procedure and Washing

The dye bath was prepared by dissolving 0.1 wt.% of Victoria Blue R dye in distilled water. Depending on the experimental design, auxiliary additives of a surfactant, electrolyte, and/or swelling agent were added to the dye bath, expressed as a mass percentage based on the solvent. Then, the dye bath liquid was transferred into vials of a combined heating/stirring system (Reacti-Therm, ThermoFisher Scientific, Waltham, MA, USA) to conduct the dyeing experiments. The Reacti-Therm system can hold up to eight vials with different dye-bath formulations and precisely control the dyeing temperature. The Reacti-Therm system was initially set at 60 °C with stirring while the samples were loaded into their respective vials, then the temperature was raised to T = 90 °C in about 30 min and held there for 1 h. Subsequently, the Reacti-Therm system was turned off, and samples were left in the vials to cool for 20 min.

After dyeing, all the samples were removed from the vials and rinsed under flowing warm tap water for 2 min followed by a cold-water rinse for another 2 min. Then, the samples were dried in a programmable convective oven starting at T = 30 °C, raising the temperature to T = 150 °C at a rate of 30 °C/min, and holding at T = 150 °C for 2 min, to fix the dye to the fabric samples. To prepare the samples for color strength analysis, the samples were washed with detergent (ECOS^®^ plus stain-fighting enzymes) to remove the oily pretreatment materials using a home-made tumbler to simulate laundering, in accordance with the protocol described in ISO standard 105-C10:2006 [19]. After the detergent washing, the samples were rinsed and dried again in the same way as described above.

### 2.4. Color Strength Analysis

The color strength of each dyed sample was quantified by measuring its spectral reflectance (R in %) in the visible range using a spectrophotometer (SPECTRO 1, Variable Inc., Chattanooga, TN, USA). The color strength was calculated at the wavelength of maximum absorbance for Victoria Blue R (λ = 615 nm) using the K/S value defined by the Kubelka–Munk equation (Equation (1)) that relates *R* with sample absorption (K) and scattering characteristics (S) [20].

(1) $$K/S=\frac{{\left(1-0.01R\right)}^{2}}{2\left(0.01R\right)}$$

The Kubelka–Munk equation is used in formulating colors for the textile, paper, and coatings industries. For these applications, it is assumed that the absorption (K) of light depends on the properties of the colorant, and the scattering (S) of a dye or pigment depends on the properties of the substrate or opacifier. The K/S value is roughly linear with respect to colorant concentration [21].

### 2.5. FTIR Analysis

To provide insight into the chemical changes occurring over the entire dyeing process, FTIR analysis was carried out on the para-aramid samples at the different stages of treatments. Attenuated total reflection Fourier transform infrared spectroscopy (ATR–FTIR) spectra were recorded using an FTIR spectrometer (Excalibur 3100, Varian Inc., Palo Alto, CA, USA) equipped with an overhead attenuated total reflection (ATR) accessory with germanium crystal (UMA 400, Varian Inc.) and a liquid nitrogen cooled mercury cadmium telluride detector. A sample was placed on a potassium bromide (KBr) plate and pressed under the germanium crystal for scanning. Each spectrum was collected within the mid-IR region from 50–4000 cm^−1^ at a resolution of 4 cm^−1^ after averaging 128 scans. The ATR spectra of samples were presented in absorbance units after taking into account the background spectrum acquired using a blank KBr plate. Between successive measurements, the germanium ATR crystal was carefully cleaned with ethanol, rinsed with distilled water, and dried to prevent cross contamination. All the spectra were ATR-corrected using Varian Resolutions Pro software (Varian Inc., Palo Alto, CA, USA).

### 2.6. SEM, Extraction Test, Tensile Strength

Scanning Electron Micrographs (SEM) of original para-aramid fibers and dyed samples were acquired on a Zeiss Auriga scanning electron microscope (Carl Zeiss SMT Inc., Oberkochen, Germany).

Complementary to the FTIR analysis, an extraction test was conducted on a set of samples dyed with AA/Soy pretreatment followed by NTP treatment, to investigate how the polymerized AA/Soy binds with the dye and para-aramids. Each sample was extracted with 40 mL of either hexane, ethanol, or 2:1 (*v*/*v*) chloroform:methanol of varying polarities at room temperature for 15 h. It was assumed that dye molecules covalently bonded to the fabric and highly polymerized AA/Soy network could not be extracted. The K/S value of each sample before and after the extraction was compared to evaluate the extent of the extraction that reduced the color strength. Furthermore, we replicated a reported novel method [22] for the fabrication of colored materials with significantly reduced dye leaching through covalent immobilization of the desired dye using plasma-generated surface radicals; this plasma dye coating procedure immobilizes a pre-adsorbed layer of a dye functionalized with a radical sensitive group on the surface through radical addition caused by a short NTP treatment. We need to point out that this study demonstrated successful dyeing of some hard-to-dye materials such as inert plastics of polyethylene and polytetrafluoroethylene but dyeing para-aramids was not attempted. We followed the same procedure of dyeing described in this reference [22] using a Disperse Red 1 acrylate dye (CAS Number 13695-46-0, Sigma-Aldrich, St. Louis, MO, USA), which is the only one used in this study that is commercially available, to dye our para-aramids. However, because our NTP source is different from that used in the referenced study, we optimized the NTP treatment time and found that a 30 s NTP time resulted in the highest K/S value (longer time would degrade the dye). Para-aramid samples dyed with the Disperse Red 1 acrylate were subjected to the same extraction procedure for comparison.

To evaluate if our pretreatment and dyeing process would affect the strength of the para-aramids, tensile testing of the para-aramid yarns was performed using a TA.XT plus texture analyzer (Stable Micro Systems, Godalming, UK), following ASTM standard [23]. Yarns of 300 Denier continuous filaments were subjected to the same pretreatment and dyeing process developed in this study, and then placed in a sealed bag to be conditioned for three days together with undyed yarns for comparison. The test was completed in triplicates and peak load at breakpoint was recorded as an indicator of the tensile strength.

## 3. Results and Discussion

### 3.1. Analysis of Dyeing Experiments

We designed Experiment A to investigate the impact of AESO concentration, the use of benzyl alcohol (Benzyl-OH) as a swelling agent, and NaCl as an electrolyte on dyeing. Furthermore, we tested the NTP treatment prior to or post AESO pretreatment in order to understand the function of NTP in improving the dyeing color strength. The design of this 4-factor, 2-level, full factorial experiment and results are presented in Table 1.

**Figure 2:**
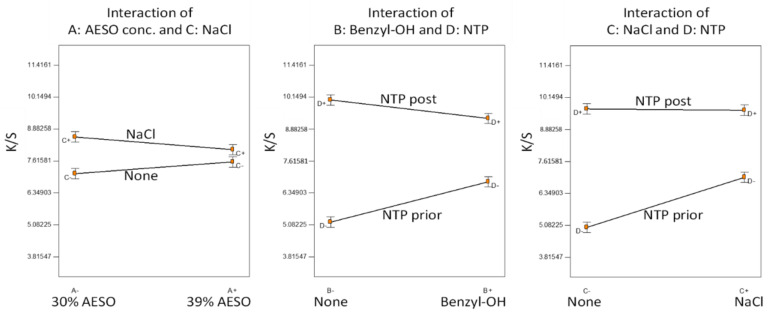
Statistical inferences of Experiment A: significant factorial interactions.

For the analysis of variance (ANOVA) of the factorial design, model term selection (including factorial interactions) was first conducted based on the half-normal probability plot, showing the factorial impact in the ascending order of (AESO conc.) < (Benzyl-OH) < (NaCl) < (NTP order). This resulted in an overall significant model (F-test, *p* < 0.0001). T-tests for each model term coefficients showed that all model terms were significant (*p* < 0.002), except for the main effect of (AESO conc.) (*p* = 0.89). This was not surprising because we did not know a suitable AESO concentration in this first experiment and randomly selected the two AESO concentrations. Since all factors were involved in significant interactions, the statistical inferences are based on the interaction plots as shown in Figure 2.

The difference between the two selected AESO concentrations was insignificant. Addition of NaCl in the dye bath slightly improved color strength, apparently when the NTP was applied prior to AESO pretreatment. The order of NTP application played an important role; NTP application post AESO pretreatment greatly increased the dyed color strength, indicating that the chemical reactions induced by NTP on AESO were the key. It appeared that the effect of NTP was confounded by the addition of benzyl alcohol and NaCl in the dye bath, so the two did not show an obvious effect if the fabrics were NTP-treated post AESO pretreatment. A reasonable explanation would be that NTP application prior to AESO pretreatment facilitated the attraction of polar NaCl and benzyl alcohol to the fabrics, while in the case of NTP application post AESO pretreatment of the fabrics was covered by AESO, and NTP was mainly used to induce reactions on the AESO.

Because the interaction between NTP and AESO was important, in Experiment B, we used a central composite design to find optimal AESO concentration and NTP treatment time post AESO pretreatment, without the addition of benzyl alcohol and NaCl in the dye bath. The detailed design and results are presented in Table 2.

Stepwise regression resulted in the selection of a reduced quadratic model as the best fit (F-test, *p* < 0.001), including only three terms of the main effect of NTP time, main effect of AESO concentration, and the interaction of the two. The quadratic terms of both NTP time and AESO concentration were not significant.

**Figure 3:**
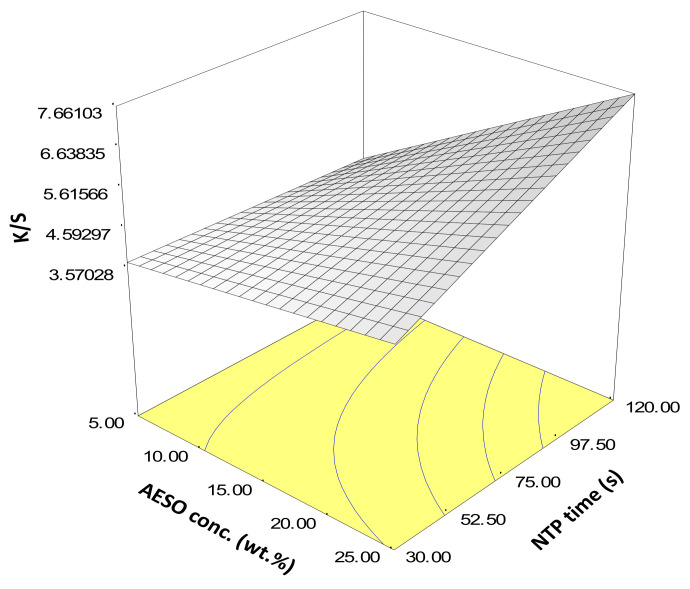
Statistical inference of Experiment B: Response surface of the central composite design.

The response surface plot for Experiment B is shown in Figure 3. Increasing both NTP time and AESO concentration increased color strength. At low AESO concentration, increasing NTP time only slightly increased color strength. However, at higher AESO concentration, increasing NTP time prominently improved color strength. This is reasonable because higher concentration of AESO and longer NTP time induced more polymerized AESO on the fabric surface that attracted more dye molecules, again corroborating the importance of AESO pretreatment followed by NTP treatment.

We also observed that AESO concentration higher than 25 wt.% resulted in an unnecessarily thick coating on the para-aramid fabrics. Therefore, we considered 25 wt.% as an optimal concentration for AESO pretreatment because the highest K/S in Experiment B was achieved at NTP time = 120 s. and AESO conc. = 25 wt.%.

In Experiment C, based on the results of Experiment A and B, we fixed AESO concentration at 25 wt.% and tested if there is any difference between just applying NTP post AESO pretreatment and applying NTP both before and after AESO pretreatment. We also slightly extended the NTP post AESO pretreatment time to 150 s, based on our observation in Figure 3. Experiment C focused on the functions of auxiliary additives of a swelling agent (benzyl alcohol), an electrolyte (NaCl), and a surfactant of Polysorbate 80 (denoted as TWEEN hereafter). The factorial design and results are shown in Table 3.

**Figure 4:**
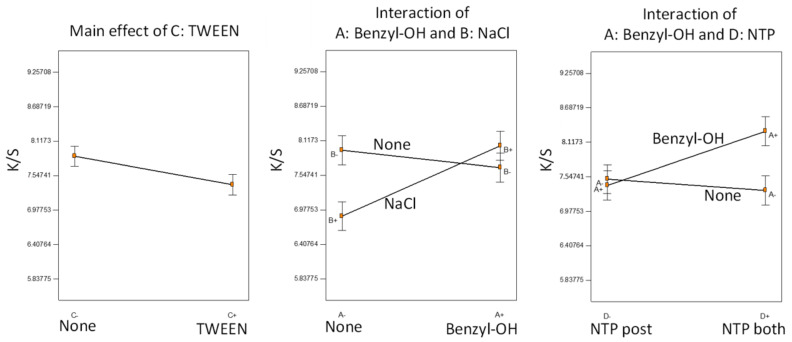
Statistical inference of Experiment C: main effect of a factor not involved in any interaction and significant interactions.

We determined an ANOVA model as the best fit (F-test, *p* < 0.0001) according to the half-normal probability plot, including all the four main factorial effects and only two interaction terms, which were statistically significant based on the *t*-test of the model coefficients (*p* < 0.05). Statistical inference is presented in Figure 4.

TWEEN was the only factor not involved in any significant interactions; the addition of the surfactant negatively affected dyeing performance, consistent with our previous observations [9,10]. The surfactant, although helping to form a dye dispersion, might also hinder the diffusion of the dye onto the para-aramid fiber fabrics. The positive effect of benzyl alcohol was significant only when the NTP was applied both before and after AESO pretreatment. Furthermore, benzyl alcohol and NaCl were confounding factors. In the absence of benzyl alcohol, the addition of NaCl lowered color strength, but with the presence of benzyl alcohol, the NaCl effect was insignificant. We may speculate that both NaCl and benzyl alcohol participated in the NTP-induced reactions with AESO, leading to different reaction pathways, and therefore, different dyeing effects. Overall, all the samples were dyed to high color strength with small variations because we fixed AESO concentration and NTP treatment time at optimal conditions. The effects of auxiliary additives were limited, compared with the major improvement of color strength caused by AESO pretreatment followed by NTP treatment.

Experiment D was conducted as a starting point to investigate the possibility of replacing AESO with a simple mixture of soybean oil and acrylic acid, because hypothetically a cross-linked network could be formed with the application of NTP to help dyeing. However, longer NTP treatment time is needed based on our preliminary trials. Furthermore, because one soybean oil molecule has an average of 4.6 double bonds and NTP could trigger self-polymerization of soybean oil [24], we tested two molar ratios of acrylic acid to soybean oil (denoted as AA/Soy hereafter) of 1.6 and 4. We also tested if the application of NTP again after dyeing would help fixing the dye. The design and results of this 3-factor, 2-level factorial experiment are shown in Table 4.

**Figure 5:**
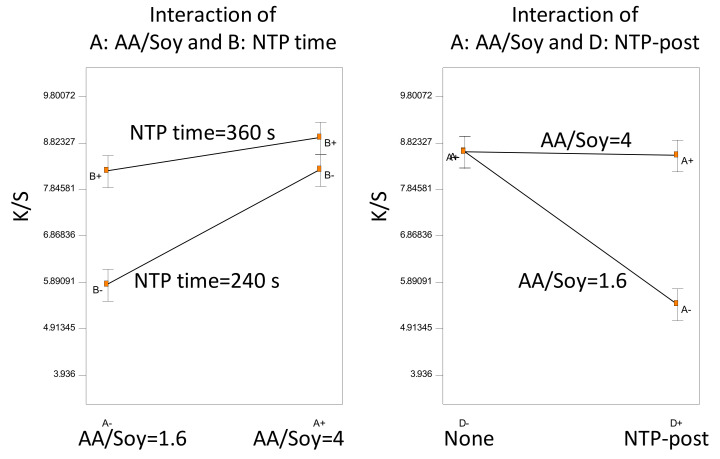
Statistical inference of Experiment D: significant factorial interactions.

Based on the half-normal probability plot, we selected an ANOVA model (F-test, *p* < 0.0001), which included all three main factors and two interaction terms (*t*-test, *p* < 0.001). Because all the factors were involved in the interactions, the statistical inference was based on the significant interaction plots as shown in Figure 5.

Overall, the color strength achieved in Experiment D is comparable with that in Experiment C using AESO, showing the potential of using AA/Soy to replace AESO. Generally, longer NTP treatment time and higher AA/Soy resulted in higher K/S values. It is interesting to observe that at AA/Soy = 1.6, a 30 s NTP treatment after dyeing greatly decreased color strength, whereas at AA/Soy = 4 there was no change. A plausible explanation would be that NTP could quickly degrade the dye molecules [10]; however, at higher AA/Soy, more chemical functionalities could absorb most NTP energy and reactive species, thus protecting the dye from degradation.

In order to evaluate if the use of auxiliary agents would help improve dyeing with the AA/Soy pretreatment, Experiment E focused on the effects of NaCl and benzyl alcohol. Furthermore, because we observed unevenly dyed samples in Experiment D due to inadequate mixing of the soybean oil and acrylic acid, the AA/Soy well dissolved in ethyl acetate (50 wt.% of AA/Soy in ethyl acetate) was used as the pretreatment solution. The design and results of this 4-factor full factorial experiment are presented in Table 5.

**Figure 6:**
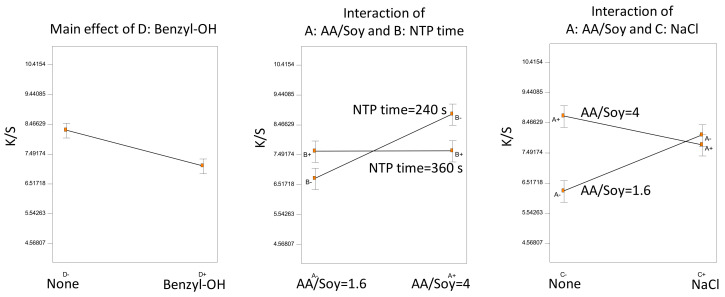
Statistical inference of Experiment E: main effect of a factor not involved in any interaction and significant interactions.

Based on the half-normal probability plot, we selected the best fit of ANOVA model (F-test, *p* < 0.0001). The statistical inference is shown in Figure 6. Only two interaction terms were significant (*t*-test, *p* < 0.01). However, the main effect of NTP treatment time was not significant (*t*-test, *p* = 0.53) due to the confounding effect of NaCl.

The swelling agent of benzyl alcohol was the only significant main factor not involved in any interactions; it negatively affected the dyeing color strength. In comparison with Experiment D, Experiment E showed similar interactions between the NTP treatment time and the AA/Soy ratio. Specifically, fixing the NTP time = 360 s, increasing AA/Soy from 1.6 to 4, did not improve color strength, while fixing NTP time = 240 s, increasing AA/Soy, significantly increased color strength.

A confounding effect of NaCl was observed. Without NaCl, higher AA/Soy yielded higher color strength; with the addition of NaCl in the dye bath, AA/Soy = 4 and AA/Soy = 1.6 resulted in statistically the same K/S. A plausible explanation would be that both NTP and NaCl would induce reactions in AA/Soy (as revealed in our FTIR analysis in Section 3.2), and the higher dosage of both would result in overreactions that decreased surface functionality for binding with the dye molecules.

Because of the kinetic nature of the reactions catalyzed by NTP and influenced by the presence of NaCl, we speculated that there exists an optimal combination of NTP treatment time, AA/Soy, and NaCl concentration. Therefore, in Experiment F, we utilized a Box–Behnken response surface design in an effort to find this optimal combination. The detailed experimental design and results are presented in Table 6.

**Figure 7:**
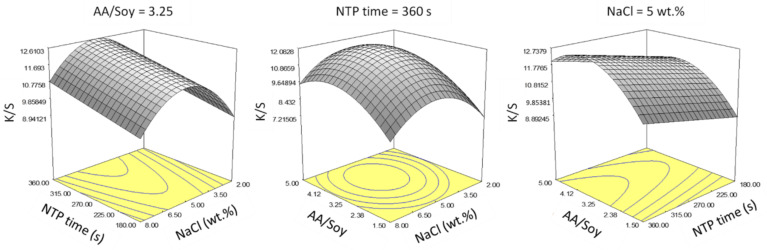
Statistical inference of Experiment F: Response surface of the Box–Behnken design.

A reduced quadratic model of response surface was selected as the best fit (F-test, *p* < 0.05) after performing stepwise regression, including the three main factor effects and the quadratic terms of only AA/Soy and NaCl concentration. Representative response surfaces are presented in Figure 7.

Within the selected ranges for the main factors, increasing NTP treatment time linearly increased color strength, so the longest NTP time = 360 s gave the best results. However, there existed an optimal point for AA/Soy and NaCl concentration. Based on the above plots, we determined an optimal condition as NTP time = 360 s, NaCl = 5% wt.%, and AA/Soy = 3.25.

**Figure 8:**
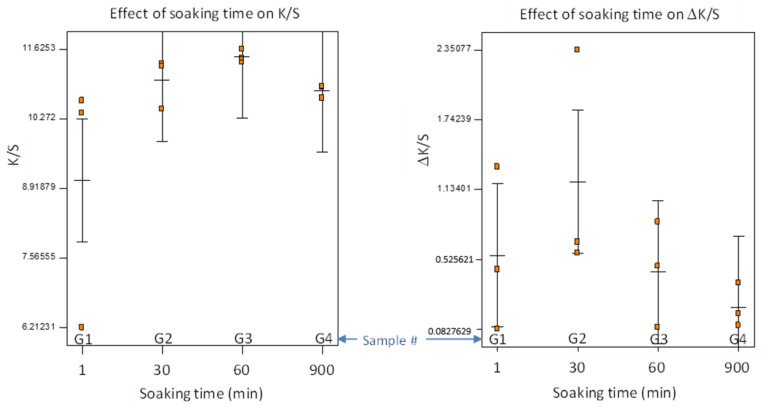
Effect of pretreatment soaking time on color strength and colorfastness (ΔK/S is defined as subtracting K/S after 2nd detergent wash from that after 1st detergent wash).

Experiment G was conducted at the determined optimal conditions, and we varied the pretreatment soaking time for practical applications, making it a one-factor factorial design with three replicates. Furthermore, all the samples in Experiment G were washed a second time with detergent by following the same protocol described in Section 2.3, and the change of K/S values (denoted as ΔK/S) was used to evaluate the colorfastness against wash. The results are presented in Figure 8, showing each data point, the means, and the least significant difference (LSD) lines.

ANOVA for the one-factor model did not detect any significant difference among the four pretreatment soaking times (F-test, *p* > 0.2), because of a large variation of K/S when the soaking time was short. There was an outlier with low K/S = 6.2, occurring with 1 min of soaking time. Generally, the soaking time appeared to affect the consistency of color strength. The ΔK/S indicated slight reduction in color strength after second detergent wash. Similarly, shorter soaking time resulted in larger variation of ΔK/S. It is recommended that a soaking time ≥60 min be used, or we need to develop a better method to evenly distribute AA/Soy into the fabrics in a shorter time (e.g., continuous soaking and pressing with rollers).

To provide a visual demonstration comparing the dyeing strengths of different treatments, images of selected samples acquired using a flatbed scanner are presented in the Supplementary File (Figure S1).

### 3.2. FTIR Analysis

To understand chemical changes during the pretreatment and dyeing processes, ATR–FTIR spectroscopy was performed at different stages of the processes. As a foundation for interpreting the FTIR spectra, Table S1 in the Supplementary File summarizes major FTIR bands of AESO, soybean oil, acrylic acid, and para-aramids.

**Figure 9:**
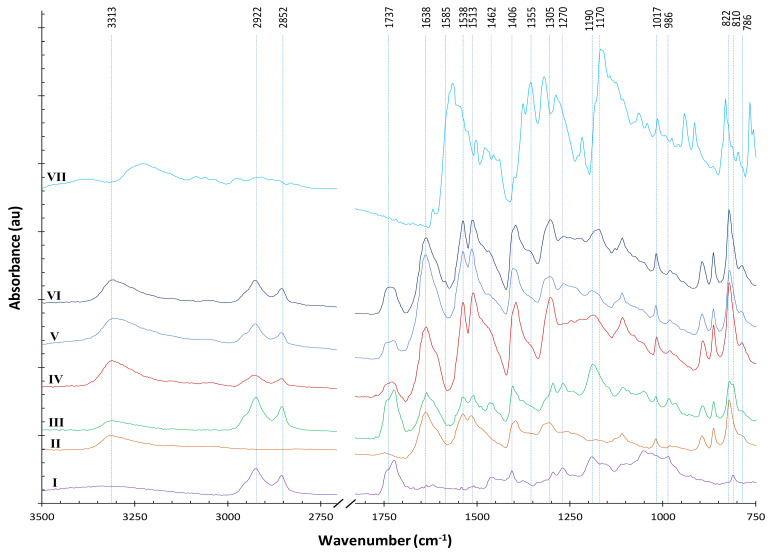
FTIR spectra at different stages of materials of (I) AESO; (II) Para-aramids; (III) Para-aramid after soaking in AESO; (IV) Para-aramid after soaking in AESO and subsequent NTP treatment; (V) dyed sample A3 pretreated with AESO without subsequent NTP treatment; (VI) dyed sample C9 pretreated with AESO with subsequent NTP treatment; (VII) Victoria Blue R.

Figure 9 presents ATR–FTIR spectra of materials at different stages of our processes using AESO for pretreatment. The AESO signal is dominated by the major peaks of soybean oil backbone (line I). A strong peak for ester carbonyl groups should appear at 1742 cm^−1^; however, due to the formation of acrylate esters, a peak should appear at nearby 1723 cm^−1^ [25]. Therefore, the peak with a deformed shape around 1737 cm^−1^ should be the overlapping of 1742 and 1723 cm^−1^. Compared with the dominant signal from soybean oil backbone, characteristics of AESO/acrylate can be found as small peaks. There are residual epoxides in the AESO that were not fully acrylated as revealed by the oxirane peaks at 1270 and 822 cm^−1^. Although major acrylate peaks (such as 1638 and 1619 cm^−1^) overlap with a strong signal from para-aramids, two distinguishable acrylate peaks at 1406 and 810 cm^−1^ can be tracked along the way of our processes to indicate the consumption of the acrylates.

For para-aramids, the bands shown in Table S1 and correspondingly in Figure 9 (line II) can be considered as an internal standard [26]. The FTIR spectrum of AESO-soaked para-aramids apparently show the addition of signals from both AESO and para-aramids (line III). After subsequent NTP treatment, changes can be seen (line IV): the acrylate peaks at 1406 and 810 cm^−1^ disappeared or significantly decreased. Although this observation was somewhat obscured by a nearby peak of para-aramids, the shapes of the two peaks more closely resemble those of the para-aramid peaks nearby, indicating the consumption of the acrylate functional groups. A sobservation can be found around 1737 cm^−1^, showing the consumption of 1723 cm^−1^ acrylate esters. The oxirane peak at 1270 cm^−1^ also disappeared (line IV). However, the signal of vinyl functional group at 986 cm^−1^ remained, albeit decreased, indicating that the AESO was not fully polymerized. The 1190 cm^−1^ peak of C–O stretching in the esters of soybean oil backbone also remained, revealing that the NTP treatment did not fully break the ester bonds.

Figure 9 also shows the FTIR spectrum of a dyed sample which was pretreated with AESO without subsequent NTP treatment (line V, sample A3, K/S = 6.14), and another dyed sample pretreated with AESO with subsequent NTP treatment (line VI, sample C9, K/S = 8.46). Other than differences at a few minor peaks, lines V and VI are very similar. The –C=O carbonyl stretching and C–O stretching in the esters at 1737 cm^−1^ and 1190 cm^−1^, respectively remained strong in lines V and VI, indicating that the NTP treatment and the dyeing process did not break much of the ester bonds, which remained after dyeing and detergent wash. However, the 1190 cm^−1^ peak in line VI shifted to the right, probably due to the influence of the dye peak at 1170 cm^−1^, because the sample of line VI was dyed to a higher K/S value. The three spectra (lines IV, V, and VI) all have a new peak emerging at 786 cm^−1^, which is in the typical region of 1,3-disubstituted or 1,2,3-trisubstituted C–H bending, meaning a possible new attachment of AESO to the para-aromatic rings. Interestingly, this new peak at 786 cm^−1^ could be generated by the NTP treatment or the thermal dyeing process, because the sample of line V was not treated with NTP after AESO soaking. In line VI, the weak absorption band at 1585 cm^−1^ was assigned to aromatic rings conjugated with α-carbonyl group, indicating that some AESO covalently bonded to para-aramid rings after NTP treatment [27].

Additionally, lines IV, V, and VI obviously have stronger FTIR signal because the AESO pretreatment, NTP treatment, and dyeing process changed the reflective index of the treated samples, enabling deeper ATR–FTIR penetration.

The FTIR spectrum of the Victoria Blue R dye is also shown in Figure 9 (line VII). Because of low uptake of the dye by the fabrics and that most of the dye peaks were obscured by the characteristic peaks of the para-aramids and AESO, no significant dye signal was detected in the dyed samples, except for that at 1355 and 1170 cm^−1^ that rendered a small shoulder in the spectra of dyed samples (lines V and VI), especially clear for the sample of line VI, which was treated with NTP and consequently dyed to a higher color strength.

**Figure 10:**
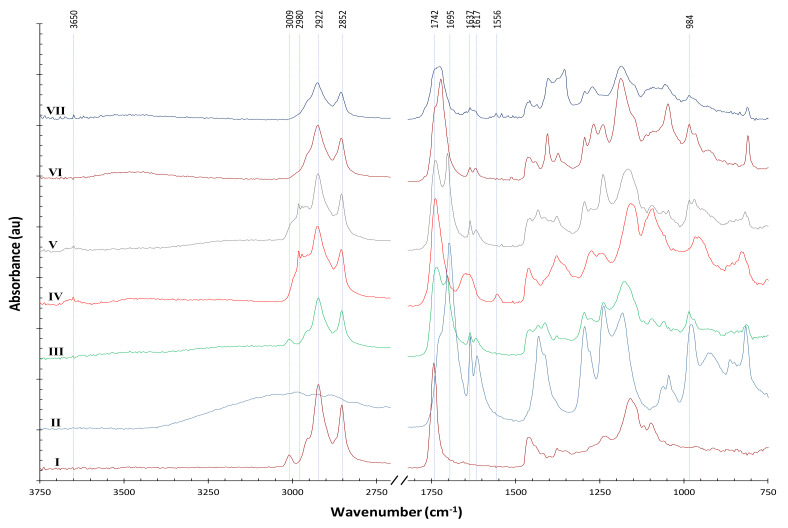
FTIR spectra at different stages of materials on KBr of (I) soybean oil; (II) acrylic acid; (III) mixture of soybean oil and acrylic acid; (IV) mixture of soybean oil and acrylic acid after 6 min NTP treatment; (V) mixture of soybean oil, acrylic acid, and NaCl; (VI) AESO; (VII) AESO after 2.5 min NTP treatment.

Because the strong signal of para-aramids obscured many meaningful peaks that reveal chemical changes, we used ATR–FTIR to monitor reactions between soybean oil and acrylic acid on KBr plates (instead of on para-aramids) in contrast to those of AESO. It is important to note that the reagents acrylic acid and AESO contain 200 or 4000 ppm of monomethyl ether of hydroquinone (MEHQ), respectively, as an inhibitor of self-polymerization. Figure 10 shows the results; line I is the spectrum of soybean oil; line II is that of acrylic acid; and line III is that of the mixture of acrylic acid and soybean oil in a molar ratio of 4:1, apparently showing the signal addition of soybean oil and acrylic acid.

After a 6 min NTP treatment of the mixture of soybean oil and acrylic acid, significant changes could be observed (line IV). A small but obvious broad peak appeared around 3650 cm^−1^, which is assigned to O–H stretching usually from alcohols or phenols. Because the NTP generated in ambient air has ozone, singlet oxygen atoms, and hydrogen peroxide as highly reactive oxidants, the unstable C=C double bonds in soybean oil could be first attacked to form epoxidized soybean oil, which could be followed by ensuing reaction with acrylic acid catalyzed by UV and/or radical species in NTP to form AESO. However, a competitive reaction could also lead to the production of polyols [28], which is evident in line IV, and the polyols could react with acrylic acid to form acrylate polymers [29,30].

After NTP treatment (line IV), the 3009 cm^−1^ peak (=C–H in soybean oil) disappeared and some new peaks appeared between 3009 and 2922 cm^−1^. The peak at 2980 cm^−1^ can be assigned to C–H (sp^3^ stretching), indicating hydrogen abstraction from the –CH_3_ or –CH_2_ of the soybean oil. However, if the FTIR spectrum was acquired on para-aramids pretreated with AA/Soy followed by NTP treatment, (see line A in Figure S2 of Supplementary File), this peak at 2980 cm^−1^ was not observed. Instead, a small peak appeared at 786 cm^−1^, which is the typical region of 1,3-disubstituted or 1,2,3-trisubstituted C–H bending and similar to the case in Figure 9 for samples pretreated with AESO. Therefore, we reasoned that the AESO or AA/Soy network was “anchored” to para-aramids at the C–H point.

The 1695 cm^−1^ peak (C=O stretching) in acrylic acid was significantly consumed with the application of NTP, and a new peak at 1556 cm^−1^ indicated nitro groups caused by the reactive nitrogen and oxygen species (RONS) in NTP; a small peak at the same wavelength can also be observed in NTP-treated AESO (line VII).

Interestingly, by simply adding NaCl to the mixture of soybean oil and acrylic acid, similar changes can be observed around 3650 and 2980 cm^−1^ (line V), indicating that NaCl triggered reactions between the soybean oil and acrylic acid at room temperature, which explains the confounding effect of NaCl we observed in Experiment E, in that the reactions with or without NaCl might lead to different kinetics and pathways. With NTP application on the mixture of soybean oil and acrylic acid (line III vs. VI), the 1637 and 1617 cm^−1^ of C=C double bonds in the acrylic acid were consumed, indicating that the NTP treatment did induce radical polymerization through the C=C double bonds. A similar observation could be found in NTP-treated AESO (line VI vs. VII). However, for the NTP-treated mixture of soybean oil and acrylic acid, the 984 cm^−1^ peak (=CH_2_) was almost completely consumed after a 6 min NTP treatment (line IV), whereas the 986 cm^−1^ peak of vinyl functional groups in AESO was still visible (line VII), indicating that a 2.5 min NTP treatment did not totally consume the vinyl groups, and longer NTP time could be applied on the AESO. The FTIR spectra of dyed samples pretreated with AESO or AA/Soy were very similar, except for some small peaks (see line B vs. C in Figure S2 of Supplementary File).

The NTP-induced chemical reactions in AESO and AA/Soy are complex, and the precise details of reaction pathways on the surface of para-aramids deserve further study. Nonetheless, based on the FTIR results, we can propose the following mechanism of improved dyeing with the pretreatment of AESO or AA/Soy. Because of the high crystallinity and inertness of para-aramids, few dye molecules can be directly adsorbed on the para-aramid surface. With the pretreatment of AESO or AA/Soy and subsequent NTP treatment, a cross-linked network (yet flexible due to the preserved long acyl chains) is formed, “anchoring” to the para-aramids with covalent bonds, and also providing functional groups that bind the dye molecules. Although the pretreatment with AESO vs. AA/Soy generated differences in the polymerized network on para-aramids, comparable dyeing effects were achieved, as evident in Section 3.1.

### 3.3. Supporting Evidence: SEM, Extraction Test, and Tensile Strength

**Figure 11:**
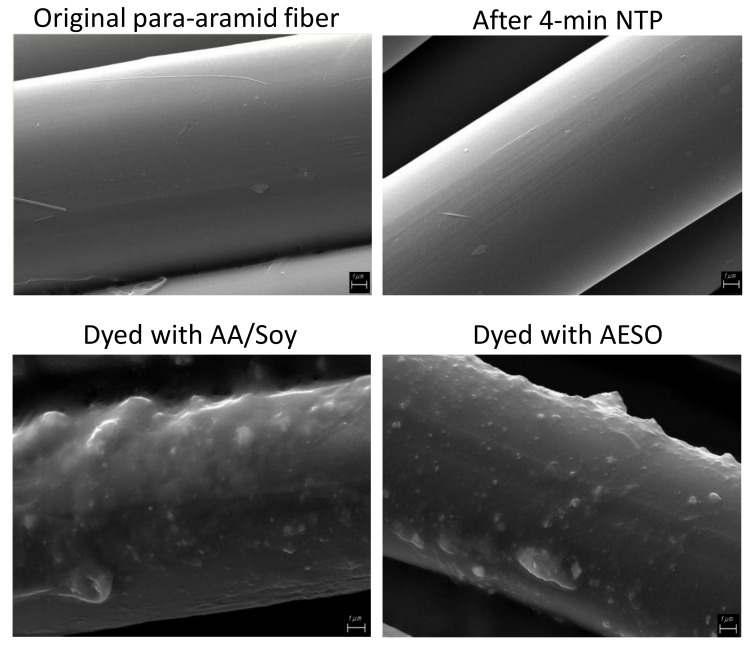
SEM micrograph of samples.

Scanning electron micrographs (SEM) reveal the smooth surface of original para-aramid fibers, and a 4 min NTP treatment did not show any visible changes on the surface (Figure 11), explaining why the NTP treatment alone would not significantly improve dyeing. The samples dyed with AESO or AA/Soy pretreatment have a resin-like coating on the para-aramid fibers, in which dye particles are embedded. It is notable that the coating survived two washes with detergent, indicating a polymerized network. Agglomeration of dye particles can also be observed, especially in the AA/Soy pretreated sample, indicating that our pretreatment procedure needs further improvement in order to evenly distribute the AESO or AA/Soy and deliver the NTP treatment.

After a 15 h extraction with three solvents of different polarity, the color strength of all the tested samples decreased. Because the K/S values were measured at different wavelengths for the blue cationic dye vs. the red disperse dye, we report here the percentage decrease in K/S value after extraction for comparison. For the samples dyed with AA/Soy pretreatment followed by NTP treatment, the K/S value decreased by 20%, 64%, and 63% after the extraction using hexane, ethanol, or 2:1 (*v*/*v*) chloroform:methanol, respectively. For the sample dyed with Disperse Red 1 acrylate, the K/S value decreased by 6%, 54%, and 84% after the extraction using hexane, ethanol, or 2:1 (*v*/*v*) chloroform:methanol, respectively. This indicates that not all the AA/Soy were polymerized (and the unpolymerized soybean oil could be easily extracted by hexane), or not all the AA/Soy were covalently bonded to the fabric. However, there was evidence that almost all the dye molecules were covalently bonded to the NTP-induced polymerized network. When the solvents were evaporated from the extractable matters and the dry lipid-dye matrices were re-dissolved in hexane, then water was added to study the partition of the dye between the hexane and water, no blue color was observed in the aqueous layer, indicating that this water-soluble dye was chemically linked to the soybean oil network.

In addition, significant color strength remained after extraction with the three solvents of different polarities, indicating a covalently bonded network formed on the surface of para-aramid fabrics, with one exception of the 84% reduction in K/S value for the sample dyed with Disperse Red 1 acrylate and extracted with chloroform:methanol, which indicates the lack of bonding between the polymerized dye and the para-aramids; the extracted sample exhibited a yellow color close to that of the undyed fabric. In this respect, the method developed in this study performed better in binding with the inert para-aramids. Furthermore, the samples dyed with Disperse Red 1 acrylate became more rigid because of the short chain of the starting monomers, while the samples dyed with AA/Soy remained flexible due to the preserved long chains of soybean oil.

It is worthwhile to mention that in dyeing textiles, dyes are usually fixed on textiles through physical entrapment or the relatively weak hydrogen bonds and van der Waals force of attraction, or stronger ionic bonds in the case of acid dye or basic dye; few dye molecules binded to textiles via covalent bonds, except for reactive dyes with cotton, wool, or polyamides such as nylon [31,32].

Tensile test results show that our developed method of dyeing para-aramids did not reduce the strength of para-aramid fibers measured as breakpoint peak load.

## 4. Conclusions

We demonstrated that deriving more functionalities of soybean oil on the surface of para-aramids further improved the dyeing color strength and colorfastness. Compared with the pretreatment using soybean oil that obtained the highest K/S value up to 3.89, pretreatment with either AESO or AA/Soy achieved K/S values higher than nine in this study. Importantly, we demonstrated through sequential experimentation that a simple mixture of AA/Soy could replace the pricy AESO to render comparable dyeing performance. NTP treatment after the AESO or AA/Soy pretreatment was essential for our developed method, because the NTP not only induced the formation of a polymerized network on the surface of para-aramids that bonded with dye molecules, but also generated covalent bonds anchoring the polymerized network to the para-aramids, which is difficult given the high crystallinity and chemical inertness of the para-aramids.

Among the auxiliary additives tested in this study, Polysorbate 80 (TWEEN) as a surfactant negatively affected the dyeing, and the effect of benzyl alcohol as a swelling agent was minimal. However, NaCl as an electrolyte showed positive effect. Therefore, we recommend an optimal formulation and condition as NTP time = 360 s (specific for the NTP source used in this study), NaCl = 5% wt.%, and AA/Soy = 3.25 for dyeing para-aramids without other chemical additives. The dyeing method developed in this study did not sacrifice the strength of para-aramids, showing the potential for this method to replace hazardous chemicals currently used in dyeing practices with renewable materials and environmentally friendly technologies, while achieving improved dyeing of para-aramid textiles for new applications.

## Figures and Tables

**Table 1:** Experiment A: factorial design and the results *.

Sample	AESO conc. (wt.%)	Benzyl-OH (wt.%)	NaCl (wt.%)	NTP Order	K/S **	Std **
A1	30	None	None	Prior	4.23	0.023
A2	30	None	None	Post	9.81	0.121
A3	30	None	8	Prior	6.14	0.075
A4	30	None	8	Post	10.53	0.505
A5	30	2	None	Prior	5.96	0.059
A6	30	2	None	Post	8.49	0.283
A7	30	2	8	Prior	7.56	0.013
A8	30	2	8	Post	10.04	0.081
A9	39	None	None	Prior	3.94	0.122
A10	39	None	None	Post	10.58	0.724
A11	39	None	8	Prior	6.48	0.191
A12	39	None	8	Post	9.22	0.318
A13	39	2	None	Prior	5.91	0.324
A14	39	2	None	Post	9.90	0.350
A15	39	2	8	Prior	7.78	0.202
A16	39	2	8	Post	8.79	0.311

* Experiment conducted at soaking time in AESO in ethyl acetate = 1 h, dye conc. = 0.1 wt.%, swelling agent = 2 wt.%, benzyl alcohol, electrolyte = 8 wt.%, NaCl, NTP treatment time = 90 s, dyeing temperature T = 90 °C, dyeing time = 1 h. ** Mean and standard error of three repeated measurements.

**Table 2:** Experiment B: central composite design and the results *.

Sample	AESO conc. (wt.%)	NTP Time (s)	K/S **	Std **
B1	0.85	75	2.51	0.086
B2	0.85	139	2.54	0.049
B3	5	30	3.63	0.032
B4	5	120	3.98	0.008
B5	15	11	3.80	0.072
B6	15	75	5.25	0.065
B7	15	75	4.65	0.127
B8	15	75	4.73	0.116
B9	15	75	5.25	0.065
B10	15	75	5.54	0.017
B11	15	75	5.77	0.104
B12	15	139	5.45	0.125
B13	25	30	5.05	0.008
B14	25	120	7.16	0.090
B15	29	11	3.55	0.016
B16	29	75	6.84	0.126

* Experiment conducted at soaking time in AESO in ethyl acetate = 15 h, dye conc. = 0.1 wt.%, dyeing temperature T = 90 °C, dyeing time = 1 h. ** Mean and standard error of three repeated measurements.

**Table 3:** Experiment C: factorial design and the results *.

Sample	Benzyl-OH (wt.%)	NaCl (wt.%)	TWEEN (wt.%)	NTP Order	K/S **	Std **
C1	None	None	None	Both	7.93	0.063
C2	None	None	None	Post	7.94	0.074
C3	None	None	3	Both	7.68	0.019
C4	None	None	3	Post	8.25	0.044
C5	None	8	None	Both	7.47	0.096
C6	None	8	None	Post	7.10	0.164
C7	None	8	3	Both	6.20	0.222
C8	None	8	3	Post	6.72	0.162
C9	2	None	None	Both	8.46	0.059
C10	2	None	None	Post	7.39	0.092
C11	2	None	3	Both	7.26	0.099
C12	2	None	3	Post	7.58	0.232
C13	2	8	None	Both	8.29	0.171
C14	2	8	None	Post	8.41	0.097
C15	2	8	3	Both	9.23	0.038
C16	2	8	3	Post	6.23	0.491

* Experiment conducted at soaking time in 25 wt.% AESO in ethyl acetate = 15 h, dye conc. = 0.1 wt.%, NTP treatment time = 150 s post AESO soaking (designated Post in Table 3) or 90 s before AESO soaking and 150 s post AESO soaking (designated Both in Table 3), dyeing temperature T = 90 °C, dyeing time = 1 h. ** Mean and standard error of three repeated measurements.

**Table 4:** Experiment D: factorial design and the results *.

Sample	AA/Soy	NTP Time (s)	Post-Dye NTP Time (s)	K/S **	Std **
D1	1.6	240	0	7.67	0.087
D2	1.6	240	30	3.99	0.054
D3	1.6	360	0	9.6	0.175
D4	1.6	360	30	6.85	0.012
D5	4	240	0	8.84	0.056
D6	4	240	30	7.66	0.039
D7	4	360	0	9.59	0.113
D8	4	360	30	8.26	0.023

* Experiment conducted at soaking time in AA/Soy mixture = 15 h, dye conc. = 0.1 wt.%, dyeing temperature T = 90 °C, dyeing time = 1 h. ** Mean and standard error of three repeated measurements.

**Table 5:** Experiment E: factorial design and the results *.

Sample	AA/Soy	NTP Time (s)	NaCl (wt.%)	Benzyl-OH (wt.%)	K/S	Std **
E1	1.6	240	None	None	6.18	0.321
E2	1.6	240	None	2	4.6	0.059
E3	4	240	None	None	10.21	0.327
E4	4	240	None	2	9.82	0.589
E5	1.6	240	5	None	8.93	0.42
E6	1.6	240	5	2	7.12	0.034
E7	4	240	5	None	8.69	0.037
E8	4	240	5	2	6.51	0.056
E9	1.6	360	None	None	6.76	0.049
E10	1.6	360	None	2	7.44	0.655
E11	4	360	None	None	7.92	0.051
E12	4	360	None	2	6.74	0.017
E13	1.6	360	5	None	9.01	0.032
E14	1.6	360	5	2	7.18	0.048
E15	4	360	5	None	8.41	0.066
E16	4	360	5	2	7.36	0.031

* Experiment conducted at soaking time in AA/Soy in ethyl acetate = 15 h, dye conc. = 0.1 wt.%, swelling agent = 2 wt.%, benzyl alcohol, electrolyte = 5 wt.%, NaCl, dyeing temperature T = 90 °C, dyeing time = 1 h. ** Mean and standard error of three repeated measurements.

**Table 6:** Experiment F: Box–Behnken design and the results *.

Sample	AA/Soy	NTP Time (s)	NaCl (wt.%)	K/S	Std **
F1	1.5	180	5	10.59	0.019
F2	1.5	270	2	5.56	0.209
F3	1.5	270	8	7.44	0.573
F4	1.5	360	5	10.50	0.387
F5	3.25	180	2	7.96	0.190
F6	3.25	180	8	9.61	0.339
F7	3.25	270	5	13.18	1.157
F8	3.25	270	5	8.94	0.052
F9	3.25	270	5	13.37	0.220
F10	3.25	270	5	12.30	0.259
F11	3.25	270	5	12.16	1.564
F12	3.25	360	2	10.97	0.126
F13	3.25	360	8	10.72	0.063
F14	5	180	5	9.62	0.044
F15	5	270	2	10.94	0.183
F16	5	270	8	9.84	0.125
F17	5	360	5	10.86	0.756

* Experiment conducted at soaking time in AA/Soy (50 wt.%) in ethyl acetate = 15 h, dye conc. = 0.1 wt.%, dyeing temperature T = 90 °C, dyeing time = 1 h. ** Mean and standard error of three repeated measurements.

## Data Availability

The data presented in this study are available on request from the corresponding author.

## Supplementary Materials

The following supporting information can be downloaded at: https://www.mdpi.com/article/10.3390/polym14030628/s1, Table S1: Characteristic FTIR bands of materials and the references; Figure S1: Scanned images of dyed samples in contrast with original undyed fabric (labels correspond to the sample numbers in Table 3, Table 4 and Table 5 and Figure 8; G4 (W2) indicates sample G4 after 2nd detergent wash); Figure S2: FTIR spectra of (A) para-aramid sample after soaking in AA/Soy and subsequent NTP treatment; (B) dyed para-aramid sample pretreated with AA/Soy with subsequent NTP treatment; (C) dyed para-aramid sample pretreated with AESO with subsequent NTP treatment.
