# Supplementary material for: Soybean Oil-Based Biopolymers Induced by Nonthermal Plasma to Enhance the Dyeing of Para-Aramids with a Cationic Dye
Source: Polymers (Basel). 2022 Feb 6;14(3):628. doi: 10.3390/polym14030628 (PMC8838635; doi:10.3390/polym14030628)
Supplement: Supplementary file 1 [file polymers-14-00628-s001.zip › polymers-1581034-supplementary.pdf]

Supplementary Materials

Soybean oil-based biopolymers induced by nonthermal plasma to enhance the dyeing of para-aramids with a cationic dye

Caleb Metzcar [b], Xiaofei Philip Ye [a*], Toni Wang [b], Christopher J. Doona [c,d]

[a] Department of Biosystems Engineering and Soil Science, The University of Tennessee, Knoxville, TN 37996, U.S.A.

[b] Department of Food Science, The University of Tennessee, Knoxville, TN 37996, U.S.A.

[c] U.S. Army Combat Capabilities Development Command – Soldier Center, Natick, MA 01760, U.S.A.

[d] Research Affiliate, Massachusetts Institute of Technology, 77 Massachusetts Ave, Cambridge, MA 02139, U.S.A.

* Corresponding author: Phone: +1-(865)-974-7129. Fax: +1-(865)-974-4514.
E-mail: xye2@utk.edu

Table S1. Characteristic FTIR bands of materials and the references.

FTIR peak (cm^{-1})	Functional group	Refs
**AESO**		
3344	–OH stretch (broad)	[1, 2]
2922	$-CH_2$ asymmetric stretch	[1, 4]
2852	$-CH_3$ symmetric stretch	[1, 4]
1462	$-CH_2$ antisymmetric deformation	[1]
1742	–C=O triglyceride carbonyl stretch	[4]
1723	acrylate esters	[7]
1638	$-CH=CH_2$ stretch of vinyl	[4, 8]
1619	C=C of acrylate group	[8, 9]
986	$-CH_2=CH(CO)-O-$ out of plane bending	[8, 10]
1406	acrylate $CH_2=CH$ scissoring of terminal alkene	[4]
1190	C–O stretch of esters	[4]
1250-1270	C–O–C stretch of oxirane	[4, 10]
822	oxirane vibration	[2, 9]
810	–C=C– rocking of acrylates	[1, 9]
**Para-aramid**		
3313	–N–H stretch	[13]
1638	amide I C=O stretch	[13]
1538	–N–H deformation	[13]
1513	amide II	[14]
1305	C–N stretch of amide III	[14]
1017, 820	C–H of para-aromatic rings	[14]

FTIR peak (cm^{-1})	Functional group	Refs
**Acrylic acid**		
1695	C=O stretch	[3]
1637	C=C stretch	[3, 5]
1432	CH_2 bending	[6]
984	$=CH_2$ out-of-plane bending	[5]
1617	C=C stretch	[6]
1297	C–C–O vibration	[6]
819	CH_2 out-of-plane bending	[6]
**Soybean oil**		
3009	=C–H stretch of aliphatic alkenes	[11, 12]
2922	$-CH_2$ asymmetric stretch	[11, 12]
2852	$-CH_3$ symmetric stretch	[11, 12]
1742	–C=O triglycerides carbonyl stretch	[11, 12]
1462	$-CH_2$ antisymmetric deformation	[11, 12]
1160	C–O stretch of esters	[11, 12]

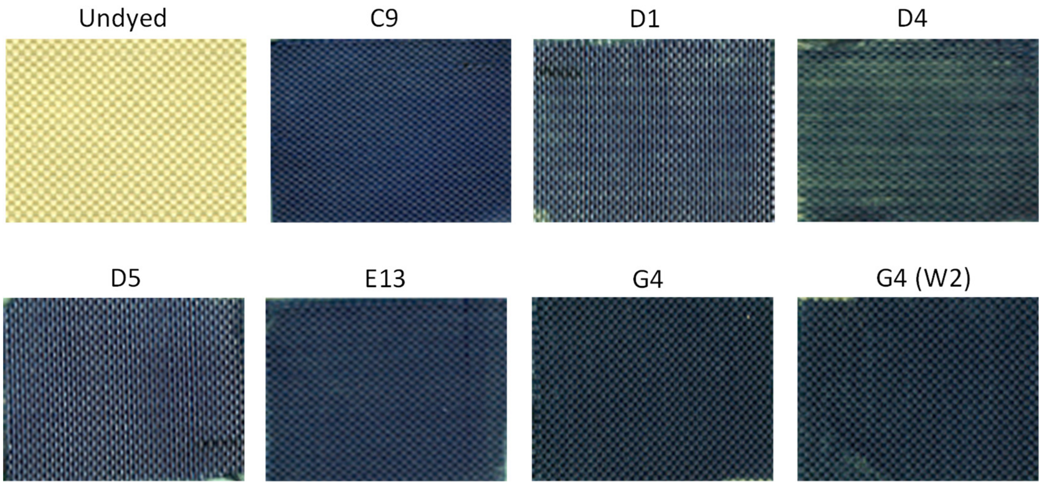

Figure S1. Scanned images of dyed samples in contrast with original undyed fabric (labels correspond to the sample numbers in Tables 3-5 and Figure 8; G4 (W2) indicates sample G4 after 2nd detergent wash)

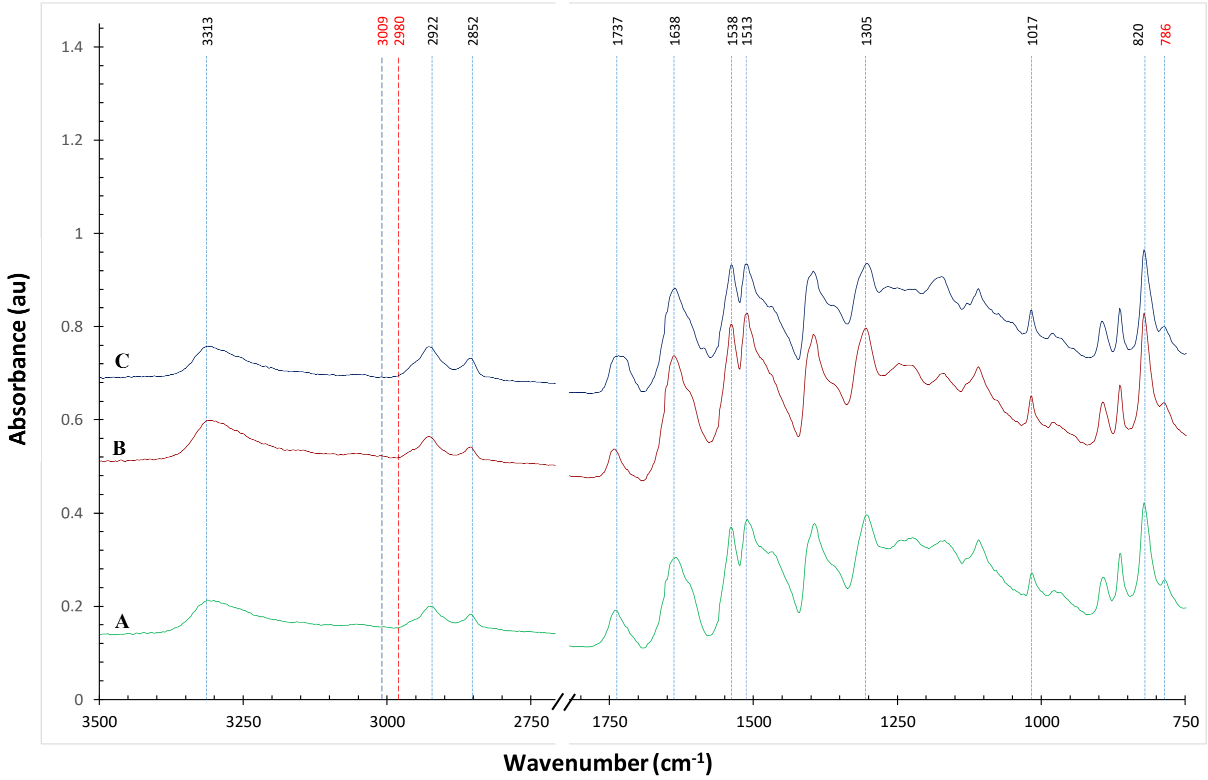

Figure S2. FTIR spectra of A) para-aramid sample after soaking in AA/Soy and subsequent NTP treatment; B) dyed para-aramid sample pretreated with AA/Soy with subsequent NTP treatment; C) dyed para-aramid sample pretreated with AESO with subsequent NTP treatment.

References

1. Fernandes, M., Souto, A.P., Gama, M., and Dourado, F., *Bacterial Cellulose and Emulsified AESO Biocomposites as an Ecological Alternative to Leather.* Nanomaterials (Basel, Switzerland), 2019. **9**(12): p. 1710.
2. Guo, A., Cho, Y., and Petrović, Z.S., *Structure and properties of halogenated and nonhalogenated soy-based polyols.* Journal of Polymer Science Part A: Polymer Chemistry, 2000. **38**(21): p. 3900-3910.
3. Berkum, S., Dee, J., Philipse, A., and Erné, B., *Frequency-Dependent Magnetic Susceptibility of Magnetite and Cobalt Ferrite Nanoparticles Embedded in PAA Hydrogel.* International journal of molecular sciences, 2013. **14**: p. 10162-10177.
4. Behera, D. and Banthia, A.K., *Synthesis, characterization, and kinetics study of thermal decomposition of epoxidized soybean oil acrylate.* Journal of Applied Polymer Science, 2008. **109**(4): p. 2583-2590.
5. Larrañaga, A., Petisco-Ferrero, S., Villanueva, R., Iturri, J., Moya, S., Meaurio, E., and Sarasua, J.-R., *Physicochemical properties of plasma polymerized acrylic acid, ε-caprolactone and lactic acid films.* Society of Plastics Engineers - EUROTEC 2011 Conference Proceedings, 2011.
6. A Umemura, J. and A Hayashi, S., *Infrared Spectra and Molecular Configurations of Liquid and Crystalline Acrylic Acids.* Bulletin of Institute for Chemical Research, Kyoto University, 1975. **52**(4): p. 585-595.
7. Esen, H. and Çayli, G., *Epoxidation and polymerization of acrylated castor oil.* European Journal of Lipid Science and Technology, 2016. **118**(6): p. 959-966.
8. Saman, N.M., Ang, D.T.C., and Gan, S.N., *Acrylated Epoxidized Soybean Oil as a Green Alternative Healant in Development of Autonomous Self-Healing Materials.* Journal of Polymers and the Environment, 2019. **27**(1): p. 118-126.
9. Li, Y. and Sun, X.S., *Synthesis and characterization of acrylic polyols and polymers from soybean oils for pressure-sensitive adhesives.* RSC Advances, 2015. **5**(55): p. 44009-44017.
10. Habib, F. and Bajpai, M., *Synthesis characterization of acrylated epoxidized soybean oil for UV-cured coatings.* Chem. Chem. Technol., 2011. **5**.
11. Zhao, X., Yang, J., Tao, D., and Xu, X., *Synthesis and Tribological Properties of Air Plasma Polymerized Soybean Oil with N-Containing Structures.* Journal of the American Oil Chemists' Society, 2014. **91**(5): p. 827-837.
12. Lumakso, F., Rohman, A., M, H., Riyanto, S., and Yusof, F., *Detection and quantification of soybean and corn oils as adulterants in avocado oil using fourier transform mid infrared (FT-MIR) spectroscopy aided with multivariate calibration.* Jurnal Teknologi, 2015. **77**(1): p. 251-255.
13. Haijuan, K., Hui, S., Jin, C., Haiquan, D., Xiaoma, D., Mengmeng, Q., Muhuo, Y., and Youfeng, Z., *Improvement of adhesion of kevlar fabrics to epoxy by surface modification with acetic anhydride in supercritical carbon dioxide.* Polymer Composites, 2019. **40**(S1): p. E920-E927.
14. Mukherjee, M., Kumar, S., Bose, S., Das, C., and Kharitonov, A., *Study on the Mechanical, Rheological, and Morphological Properties of Short Kevlar™ Fiber/s-PS Composites.* Polymer-Plastics Technology and Engineering, 2008. **47**: p. 623-629.
